# Phosphorylation of the 19S regulatory particle ATPase subunit, Rpt6, modifies susceptibility to proteotoxic stress and protein aggregation

**DOI:** 10.1371/journal.pone.0179893

**Published:** 2017-06-29

**Authors:** Esther Magdalena Marquez-Lona, Ana Lilia Torres-Machorro, Frankie R. Gonzales, Lorraine Pillus, Gentry N. Patrick

**Affiliations:** 1 Section of Neurobiology, Division of Biological Sciences, University of California San Diego, La Jolla, California, United States of America; 2 Section of Molecular Biology and UCSD Moores Cancer Center, Division of Biological Sciences, University of California San Diego, La Jolla, California, United States of America; Universitair Medisch Centrum Groningen, NETHERLANDS

## Abstract

The ubiquitin proteasome system (UPS) is a highly conserved and tightly regulated biochemical pathway that degrades the majority of proteins in eukaryotic cells. Importantly, the UPS is responsible for counteracting altered protein homeostasis induced by a variety of proteotoxic stresses. We previously reported that Rpt6, the ATPase subunit of the 19S regulatory particle (RP) of the 26S proteasome, is phosphorylated in mammalian neurons at serine 120 in response to neuronal activity. Furthermore, we found that Rpt6 S120 phosphorylation, which regulates the activity and distribution of proteasomes in neurons, is relevant for proteasome-dependent synaptic remodeling and function. To better understand the role of proteasome phosphorylation, we have constructed models of altered Rpt6 phosphorylation in *S*. *cerevisiae* by introducing chromosomal point mutations that prevent or mimic phosphorylation at the conserved serine (S119). We find that mutants which prevent Rpt6 phosphorylation at this site (*rpt6-S119A*), had increased susceptibility to proteotoxic stress, displayed abnormal morphology and had reduced proteasome activity. Since impaired proteasome function has been linked to the aggregation of toxic proteins including the Huntington’s disease (HD) related huntingtin (Htt) protein with expanded polyglutamine repeats, we evaluated the extent of Htt aggregation in our phospho-dead (*rpt6-S119A)* and phospho-mimetic (*rpt6-S119D*) mutants. We showed Htt103Q aggregate size to be significantly larger in *rpt6-S119A* mutants compared to wild-type or *rpt6-S119D* strains. Furthermore, we observed that phosphorylation of endogenous Rpt6 at S119 is increased in response to various stress conditions. Together, these data suggest that Rpt6 phosphorylation at S119 may play an important function in proteasome-dependent relief of proteotoxic stress that can be critical in protein aggregation pathologies.

## Introduction

The balance between synthesis and degradation is crucial for maintaining protein homeostasis. Many studies have shown that altered protein homeostasis occurs during normal aging and age-related disease [1–6]. In post-mitotic neurons, genetic alterations together with oxidative stress and other forms of cellular damage are thought to underlie many of the abnormalities that contribute to age-related and neurodegenerative diseases. The inability to repair or remove damaged and aggregated proteins is a key feature of the pathogenesis of many diseases of the central nervous system [7–10]. Whereas ribosomes and chaperones ensure proper synthesis and folding of proteins, the ubiquitin proteasome system (UPS) and autophagosomal/lysosomal pathways control the majority of cellular protein degradation.

The UPS is an evolutionarily conserved and tightly regulated biochemical pathway involved in protein degradation [2, 3, 11]. The selective degradation of proteins via the UPS involves three steps: recognition of the target protein via specific signals, modification of the target protein with ubiquitin chains, and delivery of the target protein to the 26S proteasome, a multi-subunit protein complex that degrades the ubiquitinated proteins [3, 12]. The 26S proteasome is a large energy-dependent protease consisting of a proteolytic 20S core particle (CP) and a 19S regulatory particle (RP). A base and a lid form the RP of the proteasome. A heterohexameric ring formed by six ATPases (Rpt1-6) constitutes part of the base, which makes direct contact with the proteolytic CP. The RP is necessary for interaction and unfolding of ubiquitinated substrates and gating of the CP to ensure proper degradation [13–15].

Accumulation of aberrant or misfolded proteins occurs in many progressive neurodegenerative disorders [8–10, 16, 17]. Alzheimer’s disease (AD), Huntington’s disease (HD), Parkinson’s disease (PD) and other clinically distinct neurodegenerative disorders are considered 'proteinopathies' of the nervous system. However, it remains unresolved whether neuronal dysfunction and disease occur as a result of toxic protein aggregation and pathogenic activities or whether protein aggregation interferes with the degradation of other proteins [18, 19]. Although numerous reports indicate a decline in proteasome function in age-related and neurodegenerative diseases (reviewed in [20, 21]), a mechanistic understanding of how proteasome activity decreases remains unclear.

We previously described the activity-dependent regulation of the 26S proteasome in mammalian neurons including the key step of phosphorylation of the 19S ATPase subunit, Rpt6, by Ca^2+^/calmodulin-dependent protein kinase II α (CaMKIIα). Additionally, we have reported a crucial role of Rpt6 phosphorylation on proteasome-dependent regulation of synaptic strength and synaptic remodeling [22–24]. *S*. *cerevisiae* has been successfully utilized as a model organism to explore aging research. Indeed, studies analyzing human protein orthologs, or expression of heterologous human disease-associated proteins have revealed the value of yeast as model to study molecular mechanisms of disease [25–29]. We confirmed that Rpt6 is phosphorylated in yeast, and that this modification is increased upon stress. In an effort to further understand the functional significance of proteasome phosphorylation, we next generated discrete models of altered Rpt6 phosphorylation and 26S proteasome function by manipulating a single residue of a single proteasome subunit in *S*. *cerevisiae*. We introduced point mutations that prevent or mimic phosphorylation at the conserved serine (S119) of Rpt6. We found that mutants which prevent Rpt6 phosphorylation at this site (*rpt6-S119A* mutants) have reduced proteasome activity, increased susceptibility to proteotoxic stress and display abnormal morphology. Since impaired proteasome function has been linked to the aggregation of toxic proteins including the HD related huntingtin (Htt) protein with expanded polyglutamine (polyQ) repeats, we evaluated the extent of Htt aggregation in our phospho-dead (*rpt6-S119A)* and phosphomimetic (*rpt6-S119D*) mutants. We showed Htt103Q aggregate size to be significantly larger in *rpt6-S119A* mutants compared to wild-type or *rpt6-S119D* mutants. Furthermore, *rpt6-S119A* mutants expressing Htt103Q displayed altered senescence profiles compared to wild-type or *rpt6-S119D* mutants. Together, these data suggest that dynamic phosphorylation of Rpt6 at S119 in yeast (S120 in mammals) may play an important, conserved function in the ability of the proteasome to counteract proteotoxic stress and protein aggregate formation.

## Materials and methods

### Yeast strains and knock-in strategy

We constructed an N-terminal 9xMyc-tagged *RPT6* strain as previously described [30]. Since *RPT6* is essential, chromosomal gene replacement was performed in a wild-type diploid strain (LPY15916, see S1 Table). To direct integration, a PCR product was prepared using oligonucleotides oLP1751 and 1752 (S3 Table) and pLP1956 (POM20) plasmid DNA as a template (S2 Table). The strain generated was LPY17011. To allow expression of the tagged protein, the *kanMX* marker was removed by Cre recombinase expression, induced from plasmid pLP192 (Life Technologies pBS39; [31]) by growth in 1% galactose. Myc-Rpt6 expression was confirmed by protein immunoblotting. The *myc-RPT6* heterozygous diploid (LPY17208) strain was sporulated and dissected to obtain LPY18040, the haploid *myc-RPT6* strain (S1 Table).

To construct the site-specific substitution mutants, an *rpt6Δ*::*kanMX* strain (LPY16270) was dissected from the yeast heterozygous diploid collection [32] using a covering plasmid containing wild-type *RPT6* (pLP2636). The *kanMX* marker of LPY16270 was replaced with a *natMX* cassette using pLP1630/p4339 (kindly provided by the C. Boone laboratory). *Apa*I digested pLP2858 and pLP2859 were used to replace *rpt6Δ*::*natMX* in LPY16270 with the *rpt6-S119A* and *rpt6-S119D* mutant versions of *5’-RPT6-kanMX-N9xMyc-RPT6-RPT6-3’*. Cre recombinase was induced as above using pLP196 (Life Technologies), and cells without the covering plasmid were recovered by selecting for growth on 5-FOA. The integrated mutants were verified by sequencing and immunoblot and subsequently backcrossed to wild-type to generate LPY19188 (*rpt6Δ*::*9myc-rpt6-S119A*) and LPY19193 (*rpt6Δ*::*9myc-rpt6-S119D*) (S1 Table).

### Plasmids

The wild-type *RPT6* gene was subcloned from a genomic tiling library [33] into pRS316 (pLP126) using the 1.8 kb *Eco*RI-*Kpn*I genomic fragment (pLP2636). A PCR product containing the *5’-RPT6-kanMX-N9xMyc-RPT6-RPT6-3’* construct from LPY17011 was cloned into TOPO (Life Technologies), generating pLP2855 (S2 Table). The construct was amplified by PCR sewing using oLP1707 + oLP883 for product one, and oLP1708 + oLP236 for product two (S3 Table). Both products were mixed and amplified with oLP1707 and 1708. The construct was verified by sequencing. The *RPT6* mutations S119A and S119D were introduced into pLP2855 by site directed mutagenesis using oligonucleotides listed in S3 Table and verified by sequencing. The resulting plasmids were pLP2858 (*rpt6-S119A*) and pLP2859 (*rpt6-S119D*) (S2 Table).

Plasmids containing the GFP-tagged N-terminal portion of the human Huntingtin protein were from Addgene (#1177, 1179 and 1180; [27]). Plasmids containing mHtt (Htt25Q, 72Q and 103Q) were transformed into *RPT6* mutant strains (LPY6496, LPY18040, LPY19188 and LPY19193) by standard methods [34].

### Immunoprecipitations and immunoblots

200 ml cultures were grown to A_600_ 0.8, collected by centrifugation and washed with 1X PBS. Pellets were lysed in 250 μL of cold IP lysis buffer (50mM Tris-HCl pH7.5, 100mM NaCl, 1mM EDTA, 0.1% Triton X-100, 10% Glycerol supplemented with Sigma COMPLETE Protease Inhibitor cocktail and 1X NME) by bead beating. Cleared lysates were incubated overnight with 25mg of mAb anti-Myc. Protein A-Sepharose (Sigma-Aldrich) previously equilibrated in IP buffer was added and samples were rotated for 4 hours at 4°C. Beads were washed 3 times with IP wash buffer (50mM Tris-HCl pH 7.5, 100mM NaCl and 1mM EDTA). Samples were boiled for 10 min in sample buffer and resolved on 10% SDS-PAGE. Proteins were transferred to a 0.2μm nitrocellulose membrane in a Bio-Rad Trans-Blot SD semi-dry transfer box, blocked in 5% milk in TBS-Tween and immunoblotted for either anti-Myc 1:4,000 (Santa Cruz, c-40; Myc1-9E10.2 hybridoma: B lymphocyte cells from Spleen: CRL-1729), pAb anti-phospho Rpt6 1:4,000 [23], β- tubulin 1:20,000 (described in [35]) or mouse anti-GFP 1:5,000 (NeuroMab, 75–131) and anti-20S antibodies (Enzo, BML-PW8195-0025).

### Growth assays

Dilution assays were performed as previously described [36] and represented five-fold serial dilutions from A_600_ 0.5 units of cells. Images were captured after 2–5 days of growth. Stress plates tested included canavanine 1μg/ml (in arg- media), ethanol 8% (in synthetic complete (SC) media), formamide 2.5% (SC), CdCl_2_ 20μM (SC) and YPD (Yeast extract-Peptone-Dextrose medium) at 37°C. Camptothecin sensitivity was assayed using 20μg/ml in DMSO, added to YPD plates buffered with 100 mM potassium phosphate to pH 7.5 [37].

### Viability assays

Assays were performed as previously described [38]. Freshly transformed strains (with pLP3012, pLP3013 or pLP3014) were grown to saturation and diluted to A_600_ 0.2 in ura- selection medium. Duplicates of 400 cells were plated on ura- plates from the same starting cultures after 1, 5, 7, 10, 12 and 15 days of growth at 30°C. Cells were counted before plating using a hemocytometer. After 3 days at 30°C, colony forming units (CFU) were counted from three independent experiments. Results were normalized to vector transformed *myc-RPT6*-wild-type strain.

### Flow cytometry

As described in [39], cells were ethanol fixed (A_600_ 0.8) during logarithmic growth. A total of 30,000 propidium iodide-stained cells of each strain were analyzed by flow cytometry (BD Accuri C6) after sonication.

### Bud profiling

Morphology was assessed by light microscopy for 2500–3000 cells per mutant strain from three independent experiments. Either single cells or cells with one bud were registered as normal. Two or more fused cells, cells with two or more buds or increased size cells when compared to wild type were registered as abnormal. Statistical analysis consisted of average and one-way ANOVA p < 0.1.

### Native-PAGE peptidase and 26S fluorogenic peptidase activity assays

Strains were grown to both A_600_ 5.0 and 0.6, spun down and freeze-dried with liquid nitrogen as previously described [40]. Briefly, cells were rehydrated using lysis buffer (25mM HEPES-KOH pH 7.4, 5mM MgCl_2_, 10% Glycerol, 1mM DTT, 2mM ATP), and ultracentrifuged at 100,000xg 30 min to obtain whole cell lysates. Native PAGE was performed as previously described [41]. 3–12% gradient gels run at constant voltage for 4 hr at 4°C were soaked in developing buffer (50mM Tris-HCl pH 7.4, 5mM MgCl_2_, 0.5mM EDTA, 1mM ATP) containing 50μM Suc-LLVY-AMC substrate. Stimulation of CP gate opening was performed using 0.02% SDS for 30 min. All images were obtained using Protein Simple FluorChem E imaging system. Gels were then transferred to nitrocellulose and probed with anti-myc and anti-20S antibodies. Chymotrypsin-like activity of 26S proteasomes from whole cell lysates was monitored over time (20 sec/ 2 hr) using Suc-LLVY-AMC substrate (Enzo) as previously described [42]. The peptidase assay was run in triplicate using a normalized protein concentration monitored on a Perkin-Elmer HTS7000 BioAssay Microplate reader. Kinetic rates from each mutant strain were plotted from the averaged triplicate data.

## Results

### Rpt6 is phosphorylated in *S*. *cerevisiae* in response to stress

Several subunits of the proteasome have been reported to be phosphorylated *in vivo* [43, 44], however, relatively few studies have addressed how phosphorylation may regulate proteasome function. Phosphorylation might regulate proteasome subunit assembly, disassembly, or distinct proteasome activities, such as peptidase activity, ATPase activity, ubiquitin binding, and/or deubiquitinating activities. Further, phosphorylation might regulate the cellular distribution and trafficking of proteasomes and association with proteasome-interacting proteins. In mammalian neurons, we found that Rpt6 is phosphorylated at serine 120 (S120) in a neuronal activity-dependent fashion [22]. It is clear that Rpt6 S120 phosphorylation increases proteasome activity and promotes the redistribution of proteasomes to synapses [22, 23, 45], although further mechanistic details remain under study.

In *S*. *cerevisiae RPT6* has been previously studied with conditional alleles in which variable phenotypes were observed, depending on the strength of each mutation [46, 47]. These studies were important for defining Rpt6 function in RP’s base assembly and its interaction with the core particle, which is indispensable for 26S proteasome assembly [48]. Similar mutants of other RP ATPases had distinct phenotypes [49], showing specialized roles in protein degradation, chromatin regulation, gene expression and DNA damage repair, among other cellular processes [50–55]. To date however, little is known about the role of phosphorylation in the regulation of RP ATPase subunits.

**Fig 1:**
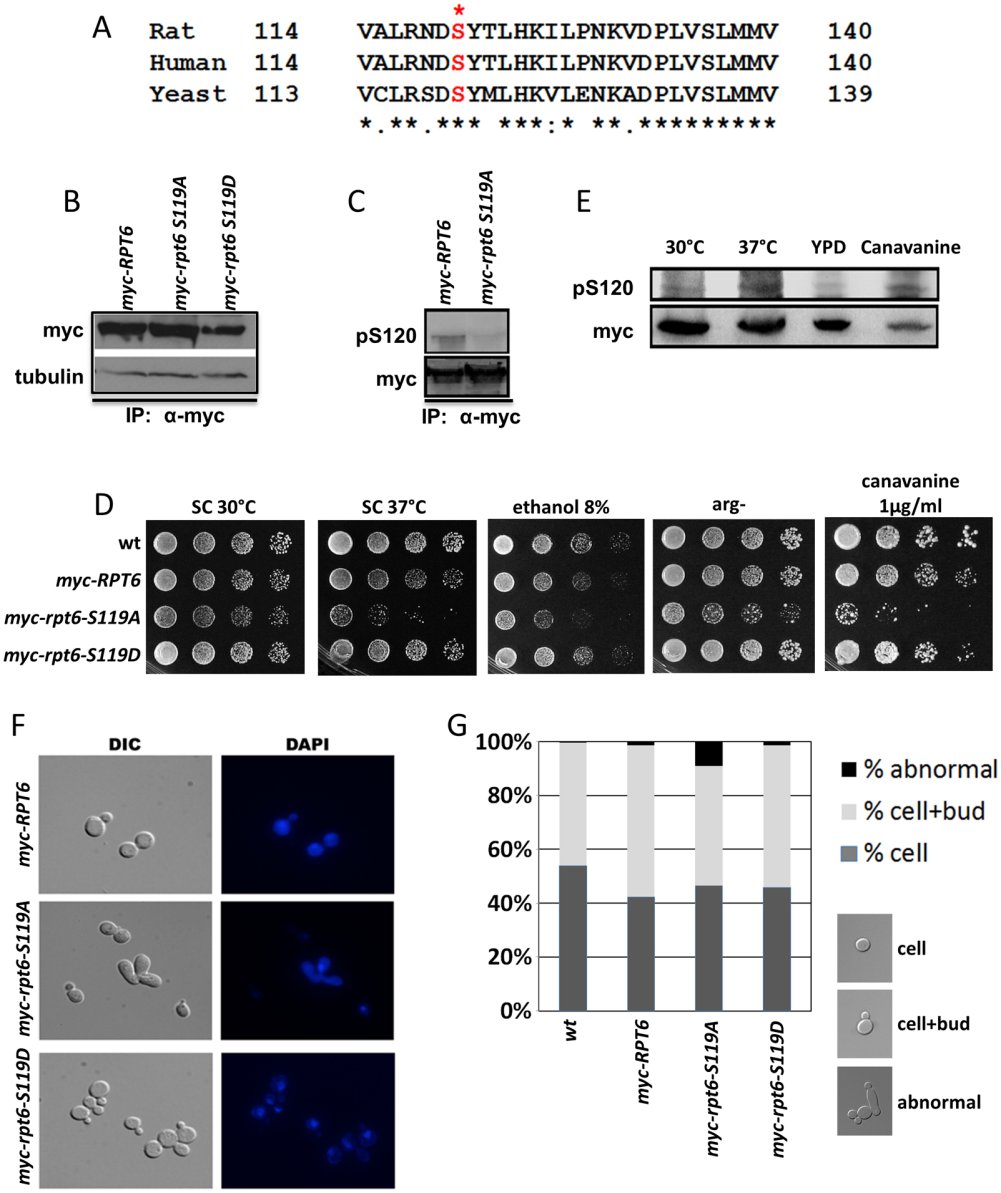
The *rpt6-S119A* mutants had increased susceptibility to proteotoxic stress and displayed abnormal morphology. (A) Alignment of Rpt6 sequences from rat, human and yeast indicate conservation of the S120 phosphorylation site. Note the presence of a serine at position 117 in yeast. This residue may contribute to some cross-immunoreactivity for the pS120 antibody in the *myc-rpt6-S119A* strain (see below). (B) Qualitative analysis of myc-tagged Rpt6 (myc-Rpt6) expression. Immunoblot depicts similar levels of expression in strains tested. (C) Representative immunoblot of *myc-RPT6* and *myc-rpt6-S119A* lysates immunoprecipitated with α-myc antibodies and resolved on SDS-PAGE. The anti-Rpt6 pS120 antibody recognizes wild-type myc-Rpt6, whereas little to no signal is observed for the *myc-rpt6-S119A* mutant, suggesting that Rpt6 is phosphorylated in yeast. (D) Increased sensitivity to proteotoxic stress is observed in the *myc*-*rpt6-S119A* mutant. The fitness of the *myc-rpt6-S119D* strain was comparable to *myc-RPT6*. The *myc-rpt6-S119A* strain was sensitive to elevated temperature, ethanol and canavanine. Arg- medium serves as a control for canavanine, which is a toxic analog of arginine. (E) Phosphorylation of endogenous Rpt6 is increased upon stress. Protein lysates from cells grown under normal and stress conditions (37° and canavanine (1ug/ml;2h)) were probed with the anti-pS120 antibody. (F) Representative micrographs of *myc-RPT6* strains (DIC and DAPI) during log phase indicate abnormal morphology in the *myc-rpt6-S119A* mutant. (G) Quantitative analysis of morphological studies in (F). Graphs denote 3 independent experiments. Abnormal morphology is increased to 10% of the total population in *myc-rpt6-S119A*.

The sequence of proteasomal subunits is conserved from yeast to humans. The sequence identities of the RP base ATPase subunits range from 66%-76%, with Rpt6 74% identical to its human ortholog [56]. Furthermore, the sequence surrounding S120 in mammals and S119 in yeast is 81% identical (Fig 1A). To address the functional relevance of Rpt6 phosphorylation on proteasome function *in vivo*, we performed studies in yeast. Two strains with chromosomally integrated mutations were constructed. The first substituted Ser119 of Rpt6 with alanine (*rpt6*-*S119A*) creating a non-modifiable phospho-dead residue; the second mutant strain was generated with an aspartic acid (*rpt6*-*S119D*) substitution as a phosphomimetic residue (S1 Table). Because the carboxy-terminus of Rpt6 contributes to proteasome assembly by establishing direct contacts with the CP [40, 57] and with protein chaperone Rpn14 [58], we constructed an N-terminal Myc-epitope tagged Rpt6 to facilitate immuno-precipitation while retaining C-terminal function. Wild-type (*myc-RPT6*) and both mutants (*myc-rpt6-S119A* and *-S119D*) were expressed from the endogenous chromosomal locus at similar levels (Fig 1B). To determine if Rpt6 was phosphorylated at S119 in yeast, Myc-Rpt6 was immunoprecipitated using a mouse anti-Myc antibody, and probed with an anti-phospho S120 Rpt6 pAb (pS120) by immunoblotting. We observed little signal to no signal for the *myc-rpt6-S119A* mutant (Fig 1C). In contrast, strong immunoreactivity was detected for the Myc-Rpt6 protein (Fig 1C) indicating that Rpt6 is phosphorylated in yeast at S119 (Fig 1C).

### The *rpt6-S119A* phospho-dead mutant is sensitive to conditions that induce protein misfolding

Proteasome function is required for maintaining protein homeostasis and cell survival during protein stress. To characterize the importance of Rpt6 phosphorylation on proteasome function, we evaluated growth of the *RPT6* phospho-mutant strains under proteotoxic stress. As shown in Fig 1D, the growth and fitness of *myc-RPT6* and the phosphomimetic mutant, *myc-rpt6-S119D*, was comparable to wild-type *RPT6* (See also S1 Fig). We did, however, find that the expression of the phosphomimetic *rpt6-S120D* on a 2μ plasmid in the *RPT6* null background promoted greater resistance to proteotoxic stress (ethanol 8%) when compared to *RPT6* or *rpt6-S120A* (also expressed on a 2μ plasmid in the *RPT6* null background) (S1 Fig). In contrast, the chromosomal *myc-rpt6-S119A* phospho-dead mutant was sensitive to growth at 37°C, to ethanol and to canavanine. The sensitivity phenotypes of the *myc-rpt6-S119A* mutant are similar to those previously found in strains with mutations in the ATPase domain of Rpt6 [40, 59]. Since growth was comparable between *myc-RPT6* and *myc-rpt6-S119D*, we suggest that phosphorylation of Rpt6 at S119 is important for maintaining proteasome function during cellular protein stress. In addition, when *myc-rpt6-S119A* was evaluated as a heterozygote, no sensitivity to proteotoxic stress was observed (S2B Fig), demonstrating that the S119A mutation in *RPT6* was recessive.

The *myc-rpt6-S119A* mutant proved to be sensitive to other agents that disrupt protein structure, including formamide and cadmium chloride (S2A Fig). Furthermore, this mutant was sensitive to the DNA damage inducing agent camptothecin (CPT), an inhibitor of topoisomerase I (S2A Fig). Collectively, these data indicate that alanine substitution at S119, preventing its posttranslational modification, results in growth defects in stress conditions similar to those of null alleles of non-essential proteasome subunits and to strains carrying thermosensitive alleles of CP or RP subunits [48, 50, 54, 55, 60].

To confirm the biological relevance of the findings above, we evaluated phosphorylation of myc-Rpt6-S119 in response to stress. When cells were grown at either high temperatures or in the presence of proteotoxic stress agents, we observed an increase in S119 phosphorylation relative to cells grown under normal conditions (Fig 1E). This suggests that phosphorylation of Rpt6 at S119 may have a role in regulating proteasome function as a response to stress mechanisms.

Because of established roles for protein turnover in cell cycle regulation [61, 62] the cell cycle profiles of both *rpt6* phospho-mutants were also analyzed. As shown in S3 Fig, a defect in progression through the G2/M phase of the cell cycle was observed in all *myc-RPT6* strains tested, which was in agreement with previous reports [46]. This shows that the N-terminal Myc-epitope may modestly interfere with Rpt6 function in the G2/M transition. This effect appears independent of any effects on proteasome function in proteotoxic and cellular stress conditions, and may thus point to a previously unsuspected role for the N-terminus. Notably, the delay in the G2/M transition was independent of S119 mutations (S3 Fig). Typically, a G2/M block is characterized by a high percentage of budded cells. All three *myc-RPT6*, *myc-rpt6-S119A* and *myc-rpt6-S119D* strains had between 40–55% budded cells (Fig 1F). However, we observed a significant increase in the multi-budded phenotype in *myc-rpt6-S119A* mutants (Fig 1G) suggesting additional defects in the control of cell division. Together, these data indicate that the *myc-rpt6-S119D* mutant strain had similar growth phenotypes to the *myc-RPT6* strain, whereas the *myc-rpt6-S119A* mutant was sensitive to proteotoxic and other cellular stressors and had an exacerbated budding defect.

### Decreased proteasome activity in the *myc-rpt6 S119A* mutant

**Fig 2:**
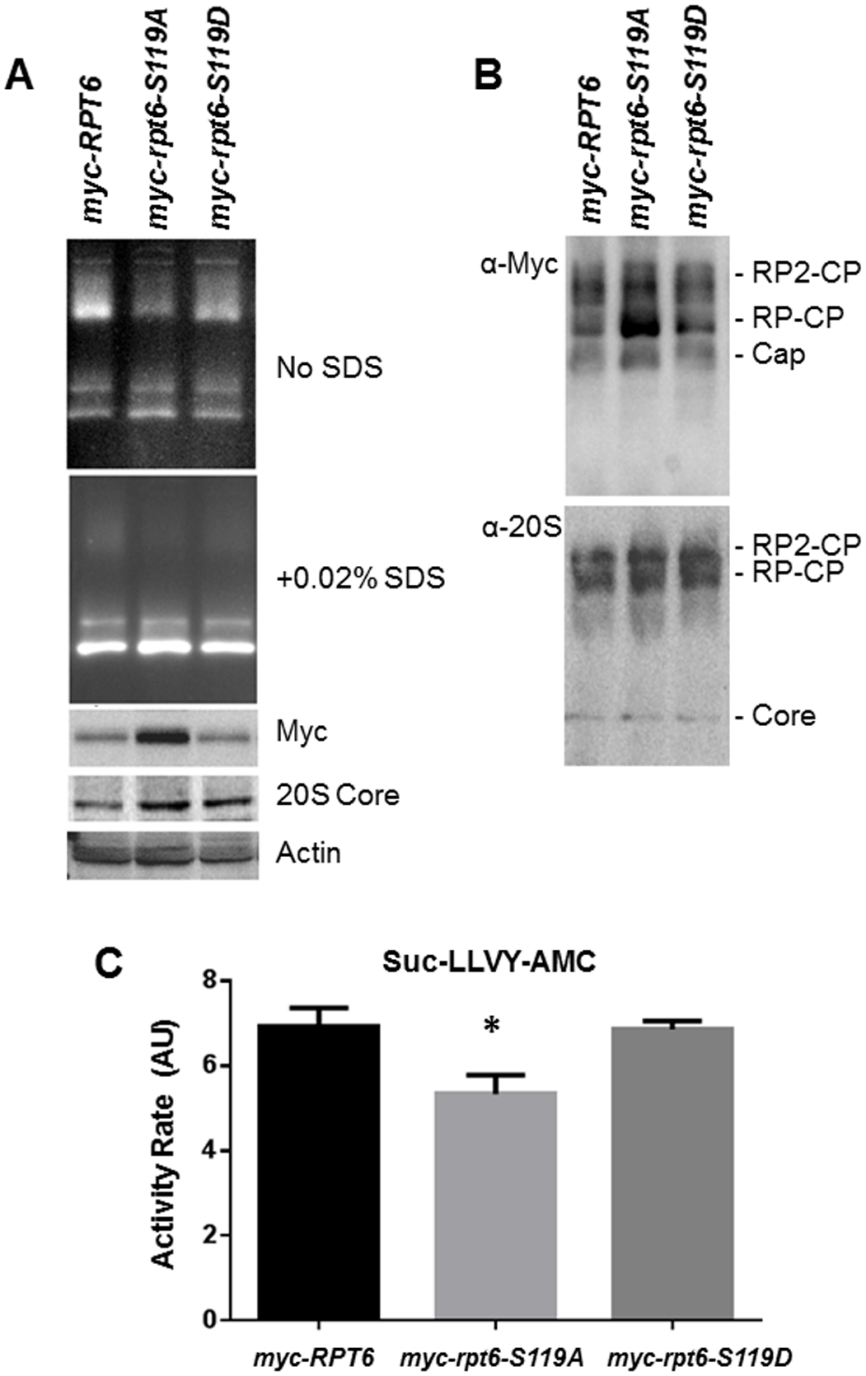
Proteasome activity was decreased in the *myc-rpt6-S119A* mutant. (A) Lysates from strains in stationary phase (A_600_>5.0) were subjected to native gel electrophoresis followed by an in-gel activity assay with the fluorogenic proteasome substrate Suc-LLVY-AMC. The *myc-rpt6-S119A* mutant displays lower activity by native in-gel analysis (before and after 20S gate opening by SDS). (B) Gels from (A) were transferred to nitrocellulose and probed with anti-myc and anti-20S antibodies. Immunoblotting indicated similar levels of proteasomes. Shown are representative in-gel and immunoblot analysis from 3 or more independent experiments. RP = Regulatory Particle, CP = Core Particle. (C) The chymotrypsin-like activity of 26S proteasomes in whole cell lysates of stationary phase cells (A_600_>5.0) was monitored by Suc-LLVY-AMC cleavage over time on microplate reader (ex. 360nm and em. 465nm). The mean ± SEM rate of Suc-LLVY-AMC cleavage is decreased in the *myc-rpt6-S119A* mutant compared to the WT and *myc-rpt6-S119D* strains. *p < 0.05, one way ANOVA. Data are from two biological samples (n = 2) with triplicate analysis.

It was important to determine if proteasome function was altered in the *myc-rpt6-S119A* mutant. We first evaluated proteasome activity from lysates of wild-type and mutant strains by native gel electrophoresis followed by an in-gel activity assay with the fluorogenic proteasome substrate Suc-LLVY-AMC. The *myc-rpt6-S119A* mutant had significantly decreased proteasome activity associated with both singly and doubly capped proteasomes (20S CP associated with one and two 19S RP, respectively), although the total level of proteasomes was comparable (Fig 2A and 2B). We additionally monitored the chymotrypsin-like activity of 26S proteasomes in whole cell lysates by Suc-LLVY-AMC cleavage over time. We found that proteasome activity was significantly decreased in lysates of *myc-rpt6-S119A* mutant when compared to *myc-RPT6* or *myc-rpt6-S119D* mutant strains (Fig 2C). We did not see enhanced proteasome activity in *myc-rpt6-S119D* mutant strain compared to *myc-RPT6*. Thus, preventing Rpt6 phosphorylation at S119 decreased proteasome function and increased susceptibility to proteotoxic stress.

### Altered Rpt6 phosphorylation exacerbates Htt polyQ aggregation and proteotoxicity

Since the cloning of the Huntington’s disease (HD) gene in 1993, transgenic models of HD have been constructed in multiple model organisms including yeast, nematodes, flies and mice [63]. *S*. *cerevisiae* has proven to be an excellent organism in which to study molecular mechanistic aspects of human neurodegenerative disease [28, 64]. In particular, HD which is caused by the expansion of a tri-nucleotide CAG repeat (poly-Q expansion) at the N-terminus of exon 1 of mutant Huntingtin (Htt), has been well-characterized [27, 65, 66]. The poly-Q expansion leads to misfolding of Htt and formation of mutant Htt-containing protein aggregates [27, 64]. Several studies have shown that proteasome activity is decreased in the affected brain regions of HD patients [67], whereas in yeast, expression of a mutant Htt fragment (Htt72Q or higher) causes aggregate formation, transcriptional dysregulation, cellular toxicity, perturbations in kynurenine pathway metabolites, increased reactive oxygen species (ROS), mitochondrial dysfunction, and defects in endocytosis and apoptotic events [27, 64–66]. Importantly, some models of HD recapitulate the relationship of mutant Htt-containing protein aggregates and decreased proteasome activity.

**Fig 3:**
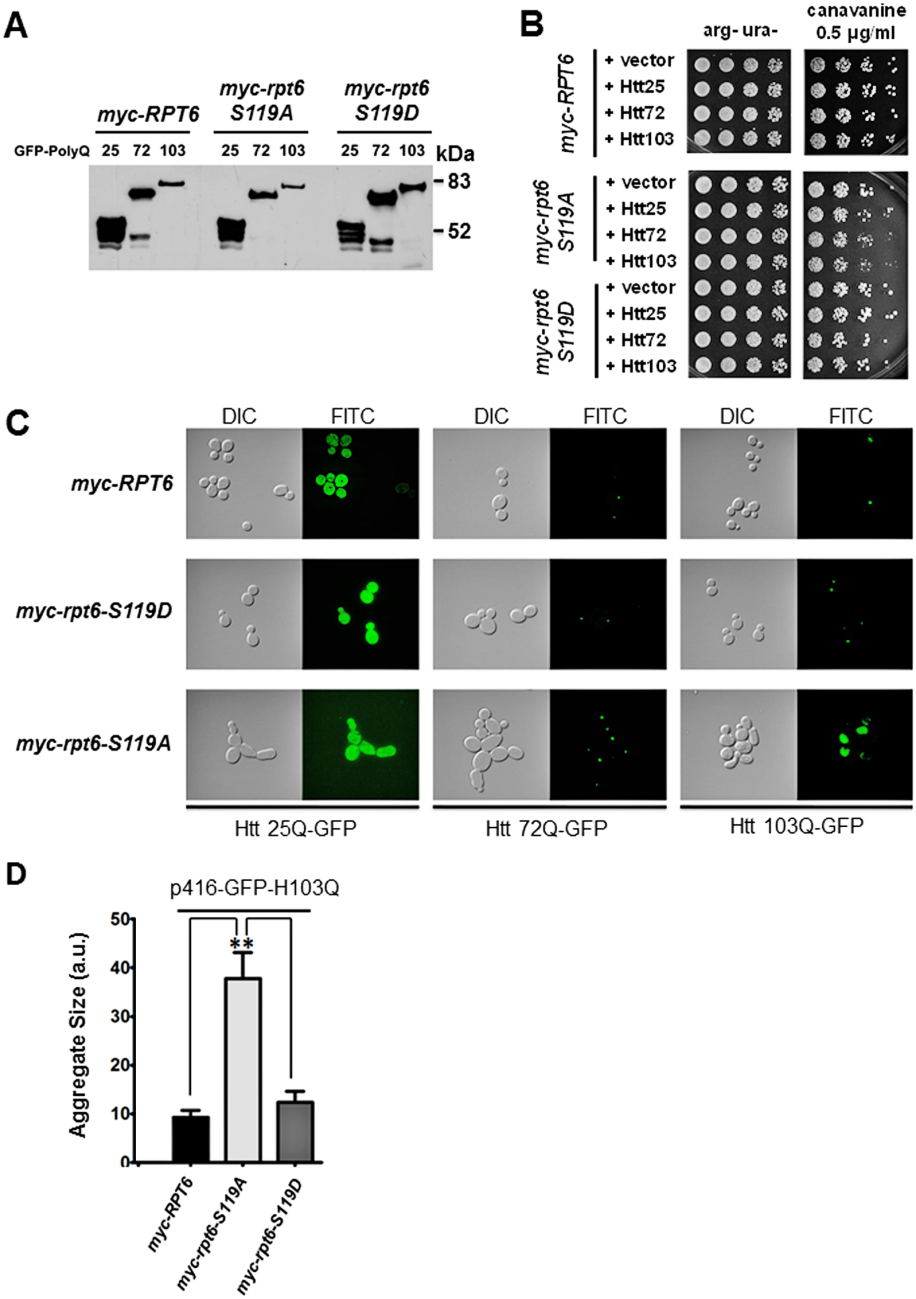
Altered Rpt6 phosphorylation and proteasome function exacerbated Htt polyQ aggregation and proteotoxicity. (A) Representative immunoblot (anti-GFP) of GFP-tagged (Htt) with different polyQ repeats (Htt25Q, Htt72Q and Htt103Q). Expression is similar in all *myc-Rpt6* strains. (B) Expression of Htt72Q and Htt103Q increased the sensitivity of *myc-rpt6-S119A* to canavanine. Strains transformed with *URA3* marked vector, Htt25, Htt72 and Htt103 plasmids were plated on canavanine to induce proteotoxic stress. The arg- ura- plates are controls. (C) Representative images of *RPT6* mutants transformed with Htt25Q, Htt72Q and Htt103Q constructs. Larger GFP-Htt aggregates are observed in the *myc-rpt6-S119A* mutant compared to WT and the *myc-rpt6-S119D* mutant. (D) Quantification of aggregate size (total GFP signal within a cell) is presented for Htt103Q expression. Graphs depict mean aggregate size ± SEM (arbitrary units). **p < 0.01, one way ANOVA. n = 45 to 60 individual GFP-aggregates (cells) measured over 2 independent experiments.

To determine if altered Rpt6 phosphorylation influences mutant Htt-induced defects in growth and the extent of protein aggregate formation, we transformed *myc-RPT6*, *myc-rpt6-S119A and myc-rpt6-S119D* strains with plasmids to induce expression of a fragment of Htt with differing polyQ lengths (Htt25Q, Htt72Q and Htt103Q) (Fig 3A). The growth of *myc*-*RPT6* and *myc-rpt6-S119D* strains expressing all mutant Htt polyQ variants upon challenge with canavanine was comparable to cells transformed with the vector control (Fig 3B). There was a slight decrease in growth with increasing length of the polyQ repeat length (Fig 3B). In contrast, the *myc-rpt6-S119A* strain displayed significantly reduced growth with increasing polyQ repeat length (Fig 3B). We subsequently evaluated protein aggregate formation of mutant GFP-Htt in all *RPT6* strains. As shown in Fig 3C, expression of Htt25Q was evenly distributed throughout the cell, whereas Htt72Q formed small protein aggregates in all strains, as previously described in a wild-type strain [27]. Htt103Q expression in the *myc-rpt6-S119A* mutant formed considerably larger mHtt aggregates compared to *myc-RPT6* and *myc-rpt6-S1119D* (Fig 3C and 3D). Together these data demonstrate that clearance of mutant Htt103Q protein aggregates is compromised in the *myc-rpt6-S119A* mutant with decreased proteasome function.

### Compromised senescence in *myc-rpt6-S119A* cells expressing Htt103Q

**Fig 4:**
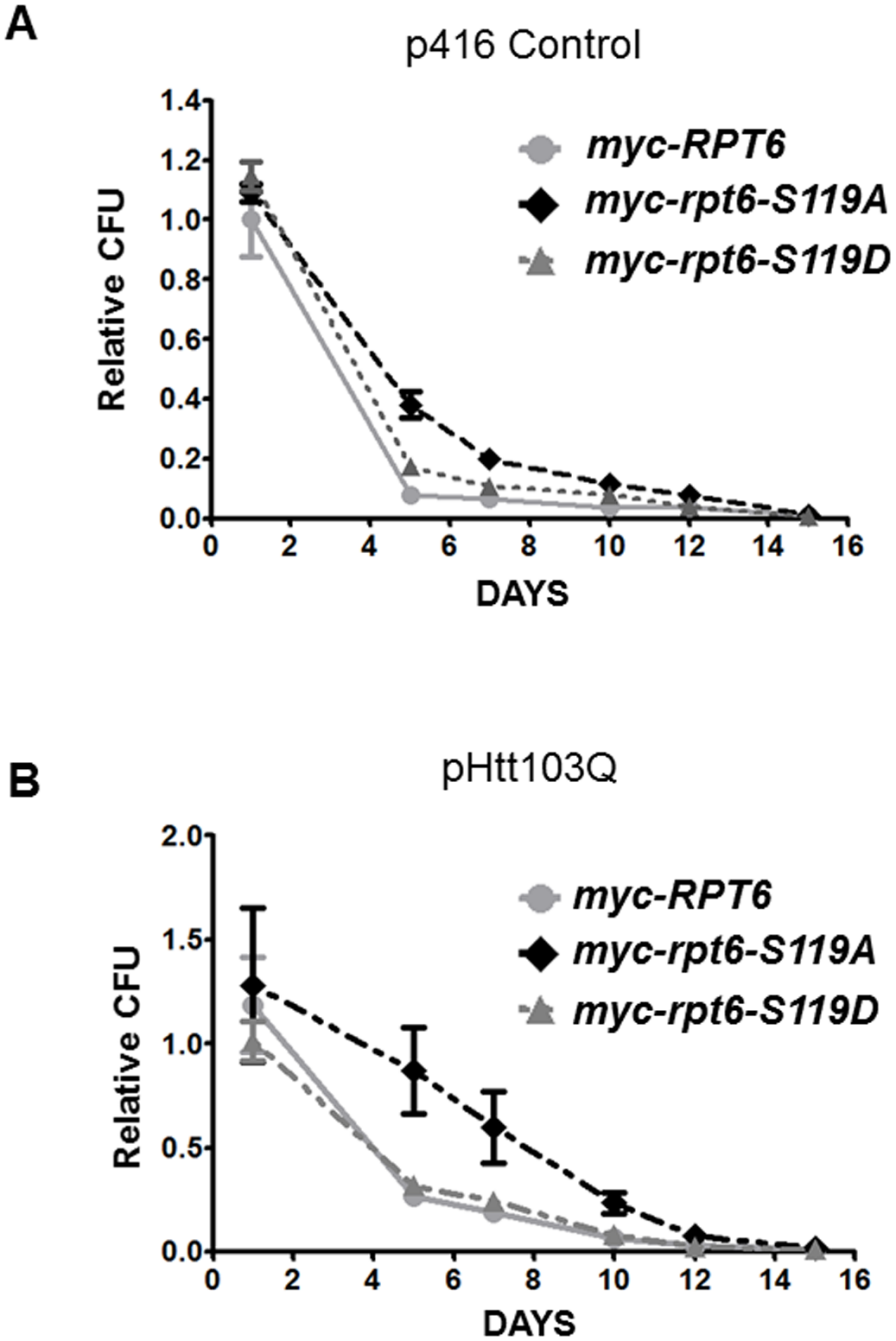
Senescent *myc-rpt6-S119A* cells expressing Htt103Q had a delayed loss of viability relative to *myc-RPT6* upon chronological aging. Strains were plated at 30°C to assess viability after 1, 5, 7, 10, 12 and 15 days of growth in liquid medium. Graphs are the average of three independent experiments performed with duplicate samples. Values were plotted relative to *myc-RPT6* transformed with vector. Comparison of all strains transformed with p416 control vector (A) or p416-Htt103Q (B). Graph for all strains expressing Htt25Q is shown in S4 Fig.

Yeast stationary phase growth or chronological life span (CLS) assays measure the survival of cell populations in post-mitotic or non-dividing phases. These metrics appear similar to those for mammalian cells such as neurons that do not divide or that have long non-mitotic rest phases [68, 69]. Because we found differences in protein aggregate size among *RPT6* strains, we sought to simulate conditions analogous to the neuronal non-dividing state by analyzing how cell survival upon extended chronological age might be affected. The viability of all *myc-RPT6* strains expressing vector control or mutant Htt103Q was measured to assess the influence of mutant Htt protein aggregation and senescence profiles. We grew all transformed strains in glucose-containing medium for up to 15 continuous days, plating on fresh medium after 1, 5, 7, 10, 12 and 15 days of growth. The colony forming units (CFU), corresponding to cells able to exit senescence and resume growth were quantified after three days and the results were normalized to *myc-RPT6* cultures transformed with vector (plated after one day of growth). The relative number of colonies indicated the survival of the strains, where the CFU number is proportional to the fitness of the strains after varying times in stationary phase. Vector-transformed *myc-RPT6*, *myc-rpt6-S119D* and *myc-rpt6-S119A* strains had a similar loss in viability after 5 days of growth, also comparable to that obtained for Rpt6 mutants transformed with Htt- 25Q (S4 Fig). However, when GFP-Htt103Q was expressed, the initial viability of all tested strains after one day of growth was 30% lower compared to vector transformants. We propose that this phenotype could reflect a problem in establishing a senescent state, delaying cell death as the cells age. This phenotype is stronger in the *myc-rpt6-S119A* mutant (Fig 4B), suggesting that normal proteasomal function may be relevant in establishing a cell’s death programming during chronological aging.

## Discussion

The UPS, which controls the majority of protein degradation in eukaryotic cells, is particularly important for counteracting altered protein homeostasis observed in age-related and neurodegenerative diseases. Indeed, the inability to rid cells of damaged proteins is believed to be key to the pathogenesis of many neurodegenerative diseases including AD, PD and HD. In some cases these proteins form heavily ubiquitinated protein aggregates. One hypothesis is that an increased proteotoxic load impairs proteasome function, thereby making cells more susceptible to stress. Alternatively, the degradation of non-toxic proteins may become impaired, which then alters normal cellular function. Historically, proteasome inhibitors have been used to understand how proteasome dysfunction contributes to disease pathogenesis. However, these have a limited utility because they are toxic to cells and cause death by apoptosis, perhaps due to cell cycle arrest [42, 70–73]. Several recent studies have reported the phosphorylation of multiple proteasome subunits. We and others have shown that the ATPase RP subunit, Rpt6, is phosphorylated at serine 120 by the CaMKIIα plasticity kinase in the mammalian CNS [22, 45]. We reported that Rpt6 phosphorylation is important for synaptic strength and proteasome-dependent remodeling of dendritic spines [23, 24]. Yet, the physiological significance of how proteasome phosphorylation regulates the rate and selectivity of substrate degradation remains an important question that is the topic of ongoing investigation.

The budding yeast *S*. *cerevisiae* has been used successfully to model neurodegenerative diseases, not only to understand the molecular mechanisms involved but also to provide insight into potential therapeutic approaches for these diseases [74]. In this study, we created phospho-dead and phospho-mimetic mutants of Rpt6 serine 119 in yeast (serine 120 in mammals) and report phosphorylation of the 26S proteasome to be an important cis-regulatory post-translational modification for the proteasome in counteracting proteotoxic stress. Using phosphospecific Rpt6 S120 antibodies we showed that Rpt6 is phosphorylated at serine 119. This posttranslational modification is increased upon temperature and proteotoxic stress, underscoring a possible role in regulation of Rpt6 and proteasome function. In addition, *rpt6-S119A* mutants that prevent Rpt6 phosphorylation at this site have reduced proteasome activity.

In mammalian cell lines and primary neurons we found that the overexpression of CaMKIIα induced a dramatic increase in the chymotryptic peptidase activity of the proteasome [22]. Furthermore, purified 26S proteasomes from Rpt6 S120A mutant mouse brains have both decreased chymotryptic activity in addition to decreased ATPase activity (F.Gonzales and G.Patrick, unpublished findings). CaMKIIα activity promotes the active sequestration of 26S proteasomes in dendritic spines [23, 45], however in this case, CaMKIIα kinase activity is dispensable which indicates that interactions of CaMKIIα with the 26S proteasomes can be sufficient to drive proteasomes into dendritic spines [45]. It is therefore plausible that in yeast the S119A mutation alters a variety of 19S proteasome activities (e.g. assembly, gating of 20S CP, substrate translocation) as well as interactions with the many proteasome interacting proteins (PIPs) [57, 58, 75–81].

Although it is yet to be determined mechanistically how the S119A mutant alters 26S proteasome function, we find that it renders the cells more susceptible to various proteotoxic stresses. One might predict that the *rpt6-S119D* mutation would confer enhanced ability to counteract proteotoxic stress in comparison to the wild-type strain. We found this to be the case when *RPT6* null strains overexpressing *rpt6-S119D* were grown in 8% ethanol (S1 Fig). When *rpt6-S119D* was expressed from the endogenous locus, the susceptibility to proteotoxic stress was similar to wild type *RPT6*. Nonetheless, while the *rpt6-S119A* strain grew similar to wild type and *rpt6-S119D* in normal growth media, growth was significantly perturbed in stress-inducing conditions. As we demonstrated that the myc-Rpt6-S119A protein (the only cellular source of the Rpt6 protein in the null background) is clearly incorporated into functional proteasomes allowing viability of the cells in normal growth media, we suggest that Rpt6 phosphorylation has a role in the cellular response to proteotoxic stress.

We also found protein aggregates of mutant Huntingtin protein with expanded CAG repeats (mHtt103Q) to be significantly larger in *rpt6-S119A* mutants compared to wild-type or *rpt6-S119D* mutants. Intriguingly, Krobitsch and Lindquist did not find any effects on aggregate size in various partial loss-of-function mutations of the UPS, including mutations in genes encoding 20S (*DOA3*) and 26S (*SEN3/RPN2*) proteasome subunits [27]. In this work, we showed that Htt aggregates did not appear to be toxic for cells unless sensitized by proteotoxic stress, such as growth in canavanine. Furthermore, Lin et al. (2013) showed that expressing Rpt6 S120A in mouse brain blocked the ability of a cAMP-elevating reagent to enhance proteasome activity, whereas the phosphomimetic Rpt6 mutant (Rpt6 S120D) increased proteasome activity, reduced HTT aggregates, and ameliorated motor impairment [82]. This further supports the idea that Rpt6 S120 phosphorylation is important for proteasome function under various stress conditions including protein aggregation.

We expected *myc-rpt6-S120A* mutants, which are significantly more sensitive to proteotoxic stress and have larger toxic Htt103Q aggregates (Figs 1 thru 3), to be more susceptible to chronological aging. However, viability was instead increased in *myc-rpt6-S120A* mutant strain compared to *myc-rpt6* and *myc-rpt6-S119D* strains (Fig 4B). In part, this is reminiscent of the findings in neurodegenerative disease models, where persistence of a non-functional cell state can exhaust brain resources, causing longer time in a senescent state, until apoptosis is activated [83–85]. This result will be of interest to pursue as the possibility of an adaptive response involving activation of chaperones or autophagy remains. It is also possible that the senescence phenotypes observed here are distinct from the response to stress discussed above.

Together, our findings support the notion that Rpt6 phosphorylation at S119/S120 plays an important, conserved function in the ability of the proteasome to counteract proteotoxic stress and protein aggregate formation. Interestingly, the proteasome has been shown to be modified by other post-translational modifications [86–89, 89, 90]. Future studies will be important to help determine the mechanisms of how Rtp6 phosphorylation or other modifications alter 26S proteasome function in both normal and pathological states.

## Supporting information

S1 Fig Overexpression of phosphomimetic *rpt6-S120D* promotes resistance to proteotoxic stress in the *RPT6* null background. *rpt6Δ* null strains overexpressing *RPT6* or *rpt6-S120A* were sensitive to ethanol 8%. All plasmids transformed in the experiment were 2μ.
(PDF)

S2 Fig The *rpt6-S119A* mutant had increased susceptibility to proteotoxic stress. (A) Growth assays for all mutants challenged with elevated temperature or protein instability and DNA repair blocking agents. (B) WT and mutant strains were mated to WT yeast to produce diploids. Heterozygous diploid strains (*) had comparable growth to WT diploid yeast indicating that the *rpt6* mutants are not dominant.
(PDF)

S3 Fig Cell cycle profiles of *myc-RPT6*, *myc-rpt6-S119A*, *and myc-rpt6-S119D* strains were comparable. Flow cytometry analysis performed during logarithmic phase A_600_ 0.6 (30°C and 34°C) and stationary phase A_600_ >5 (30°C) revealed a similar delay in cell cycle progression in all myc-tagged strains.
(PDF)

S4 Fig Strains *myc-rpt6-S119A* and *myc-rpt6-S119D* expressing Htt25Q had a similar loss of viability upon chronological aging. Strains were grown in liquid ura- medium at 30°C and plated to asses viability after 1, 5, 7, 10, 12 and 15 days of growth. The graph is the average of three independent experiments performed with duplicate samples. Values were plotted relative to *myc-RPT6* transformed with vector.
(PDF)

S1 Table List of strains utilized in this study. (PDF)

S2 Table List of plasmids utilized in this study. (PDF)

S3 Table List of oligonucleotides utilized in this study. (PDF)

## References

[1] Matthews W, Driscoll J, Tanaka K, Ichihara A, Goldberg AL. Involvement of the proteasome in various degradative processes in mammalian cells. Proc Natl Acad Sci U S A. 1989; 86: 2597–2601. 2539595 10.1073/pnas.86.8.2597 PMC286964

[2] Jentsch S. Ubiquitin-dependent protein degradation: a cellular perspective. Trends Cell Biol. 1992; 2: 98–103. 14732013 10.1016/0962-8924(92)90013-d

[3] Ciechanover A. The ubiquitin-proteasome proteolytic pathway. Cell. 1994; 79: 13–21. 7923371 10.1016/0092-8674(94)90396-4

[4] Muller S, Schwartz LM. Ubiquitin in homeostasis, development and disease. Bioessays. 1995; 17: 677–684. doi: 10.1002/bies.950170804 7661849 10.1002/bies.950170804

[5] Stolzing A, Grune T. The proteasome and its function in the ageing process. Clin Exp Dermatol. 2001; 26: 566–572. 11696059 10.1046/j.1365-2230.2001.00867.x

[6] Goldberg AL, St John AC. Intracellular protein degradation in mammalian and bacterial cells: Part 2. Annu Rev Biochem. 1976; 45: 747–803. doi: 10.1146/annurev.bi.45.070176.003531 786161 10.1146/annurev.bi.45.070176.003531

[7] Keller JN, Gee J, Ding Q. The proteasome in brain aging. Ageing Res Rev. 2002; 1: 279–293. 12039443 10.1016/s1568-1637(01)00006-x

[8] Bossy-Wetzel E, Schwarzenbacher R, Lipton SA. Molecular pathways to neurodegeneration. Nat Med. 2004; 10 Suppl: S2–S9. 15272266 10.1038/nm1067

[9] Nijholt DA, De KL, Elfrink HL, Hoozemans JJ, Scheper W. Removing protein aggregates: the role of proteolysis in neurodegeneration. Curr Med Chem. 2011; 18: 2459–2476. 21568912 10.2174/092986711795843236

[10] Vilchez D, Saez I, Dillin A. The role of protein clearance mechanisms in organismal ageing and age-related diseases. Nat Commun. 2014; 5: 5659 doi: 10.1038/ncomms6659 25482515 10.1038/ncomms6659

[11] Schwartz AL, Ciechanover A. Ubiquitin-mediated protein modification and degradation. Am J Respir Cell Mol Biol. 1992; 7: 463–468. doi: 10.1165/ajrcmb/7.5.463 1329865 10.1165/ajrcmb/7.5.463

[12] Jana NR. Protein homeostasis and aging: role of ubiquitin protein ligases. Neurochem Int. 2012; 60: 443–447. doi: 10.1016/j.neuint.2012.02.009 22353631 10.1016/j.neuint.2012.02.009

[13] Hershko A, Ciechanover A. The ubiquitin system. Annu Rev Biochem. 1998; 67: 425–479. doi: 10.1146/annurev.biochem.67.1.425 9759494 10.1146/annurev.biochem.67.1.425

[14] Ciechanover A. Tracing the history of the ubiquitin proteolytic system: the pioneering article. Biochem Biophys Res Commun. 2009; 387: 1–10. doi: 10.1016/j.bbrc.2009.06.065 19539608 10.1016/j.bbrc.2009.06.065

[15] Pickart CM, Cohen RE. Proteasomes and their kin: proteases in the machine age. Nat Rev Mol Cell Biol. 2004; 5: 177–187. doi: 10.1038/nrm1336 14990998 10.1038/nrm1336

[16] Mayer RJ. From neurodegeneration to neurohomeostasis: the role of ubiquitin. Drug News Perspect. 2003; 16: 103–108. 12792671 10.1358/dnp.2003.16.2.829327

[17] Berke SJ, Paulson HL. Protein aggregation and the ubiquitin proteasome pathway: gaining the UPPer hand on neurodegeneration. Curr Opin Genet Dev. 2003; 13: 253–261. 12787787 10.1016/s0959-437x(03)00053-4

[18] Bence NF, Sampat RM, Kopito RR. Impairment of the ubiquitin-proteasome system by protein aggregation. Science. 2001; 292: 1552–1555. doi: 10.1126/science.292.5521.1552 11375494 10.1126/science.292.5521.1552

[19] Wang J, Wang CE, Orr A, Tydlacka S, Li SH, Li XJ. Impaired ubiquitin-proteasome system activity in the synapses of Huntington's disease mice. J Cell Biol. 2008; 180: 1177–1189. doi: 10.1083/jcb.200709080 18362179 10.1083/jcb.200709080 PMC2290845

[20] Tanaka K, Matsuda N. Proteostasis and neurodegeneration: the roles of proteasomal degradation and autophagy. Biochim Biophys Acta. 2014; 1843: 197–204. doi: 10.1016/j.bbamcr.2013.03.012 23523933 10.1016/j.bbamcr.2013.03.012

[21] Dantuma NP, Bott LC. The ubiquitin-proteasome system in neurodegenerative diseases: precipitating factor, yet part of the solution. Front Mol Neurosci. 2014; 7: 70 doi: 10.3389/fnmol.2014.00070 25132814 10.3389/fnmol.2014.00070 PMC4117186

[22] Djakovic SN, Schwarz LA, Barylko B, DeMartino GN, Patrick GN. Regulation of the proteasome by neuronal activity and calcium/calmodulin-dependent protein kinase II. J Biol Chem. 2009; 284: 26655–26665. doi: 10.1074/jbc.M109.021956 19638347 10.1074/jbc.M109.021956 PMC2785353

[23] Djakovic SN, Marquez-Lona EM, Jakawich SK, Wright R, Chu C, Sutton MA et al Phosphorylation of Rpt6 regulates synaptic strength in hippocampal neurons. J Neurosci. 2012; 32: 5126–5131. doi: 10.1523/JNEUROSCI.4427-11.2012 22496558 10.1523/JNEUROSCI.4427-11.2012 PMC3348785

[24] Hamilton AM, Oh WC, Vega-Ramirez H, Stein IS, Hell JW, Patrick GN et al Activity-dependent growth of new dendritic spines is regulated by the proteasome. Neuron. 2012; 74: 1023–1030. doi: 10.1016/j.neuron.2012.04.031 22726833 10.1016/j.neuron.2012.04.031 PMC3500563

[25] Braun RJ. Ubiquitin-dependent proteolysis in yeast cells expressing neurotoxic proteins. Front Mol Neurosci. 2015; 8: 8 doi: 10.3389/fnmol.2015.00008 25814926 10.3389/fnmol.2015.00008 PMC4357299

[26] Khurana V, Lindquist S. Modelling neurodegeneration in *Saccharomyces cerevisiae*: why cook with baker's yeast? Nat Rev Neurosci. 2010; 11: 436–449. doi: 10.1038/nrn2809 20424620 10.1038/nrn2809

[27] Krobitsch S, Lindquist S. Aggregation of huntingtin in yeast varies with the length of the polyglutamine expansion and the expression of chaperone proteins. Proc Natl Acad Sci U S A. 2000; 97: 1589–1594. 10677504 10.1073/pnas.97.4.1589 PMC26479

[28] Pereira C, Bessa C, Soares J, Leao M, Saraiva L. Contribution of yeast models to neurodegeneration research. J Biomed Biotechnol. 2012; 2012: 941232 doi: 10.1155/2012/941232 22910375 10.1155/2012/941232 PMC3403639

[29] Hieter P, Boycott KM. Understanding rare disease pathogenesis: a grand challenge for model organisms. Genetics. 2014; 198: 443–445. doi: 10.1534/genetics.114.170217 25316782 10.1534/genetics.114.170217 PMC4196600

[30] Gauss R, Trautwein M, Sommer T, Spang A. New modules for the repeated internal and N-terminal epitope tagging of genes in *Saccharomyces cerevisiae*. Yeast. 2005; 22: 1–12. doi: 10.1002/yea.1187 15565729 10.1002/yea.1187

[31] Sauer B. Recycling selectable markers in yeast. Biotechniques. 1994; 16: 1086–1088. 8074874

[32] Winzeler EA, Shoemaker DD, Astromoff A, Liang H, Anderson K, Andre B et al Functional characterization of the *S*. *cerevisiae* genome by gene deletion and parallel analysis. Science. 1999; 285: 901–906. 10436161 10.1126/science.285.5429.901

[33] Jones GM, Stalker J, Humphray S, West A, Cox T, Rogers J et al A systematic library for comprehensive overexpression screens in *Saccharomyces cerevisiae*. Nat Methods. 2008; 5: 239–241. doi: 10.1038/nmeth.1181 18246075 10.1038/nmeth.1181

[34] Elble R. A simple and efficient procedure for transformation of yeasts. Biotechniques. 1992; 13: 18–20. 1503765

[35] Bond JF, Fridovich-Keil JL, Pillus L, Mulligan RC, Solomon F. A chicken-yeast chimeric beta-tubulin protein is incorporated into mouse microtubules in vivo. Cell. 1986; 44: 461–468. 3753663 10.1016/0092-8674(86)90467-8

[36] Chang CS, Pillus L. Collaboration between the essential Esa1 acetyltransferase and the Rpd3 deacetylase is mediated by H4K12 histone acetylation in S*accharomyces cerevisiae*. Genetics. 2009; 183: 149–160. doi: 10.1534/genetics.109.103846 19596907 10.1534/genetics.109.103846 PMC2746140

[37] Nitiss J, Wang JC. DNA topoisomerase-targeting antitumor drugs can be studied in yeast. Proc Natl Acad Sci U S A. 1988; 85: 7501–7505. 2845409 10.1073/pnas.85.20.7501 PMC282219

[38] Reifsnyder C, Lowell J, Clarke A, Pillus L. Yeast SAS silencing genes and human genes associated with AML and HIV-1 Tat interactions are homologous with acetyltransferases. Nat Genet. 1996; 14: 42–49. doi: 10.1038/ng0996-42 8782818 10.1038/ng0996-42

[39] Chang CS, Clarke A, Pillus L. Suppression analysis of esa1 mutants in *Saccharomyces cerevisiae* links NAB3 to transcriptional silencing and nucleolar functions. G3 (Bethesda). 2012;2: 1223–1232. 23050233 10.1534/g3.112.003558 PMC3464115

[40] Park S, Kim W, Tian G, Gygi SP, Finley D. Structural defects in the regulatory particle-core particle interface of the proteasome induce a novel proteasome stress response. J Biol Chem. 2011; 286: 36652–36666. doi: 10.1074/jbc.M111.285924 21878652 10.1074/jbc.M111.285924 PMC3196138

[41] Elsasser S, Schmidt M, Finley D. Characterization of the proteasome using native gel electrophoresis. Methods Enzymol. 2005; 398: 353–363. doi: 10.1016/S0076-6879(05)98029-4 16275342 10.1016/S0076-6879(05)98029-4

[42] Kisselev AF, Goldberg AL. Monitoring activity and inhibition of 26S proteasomes with fluorogenic peptide substrates. Methods Enzymol. 2005; 398: 364–378. doi: 10.1016/S0076-6879(05)98030-0 16275343 10.1016/S0076-6879(05)98030-0

[43] Etlinger JD, Li SX, Guo GG, Li N. Phosphorylation and ubiquitination of the 26S proteasome complex. Enzyme Protein. 1993; 47: 325–329. 7697130 10.1159/000468690

[44] Rivett AJ, Bose S, Brooks P, Broadfoot KI. Regulation of proteasome complexes by gamma-interferon and phosphorylation. Biochimie. 2001;83: 363–366. 11295498 10.1016/s0300-9084(01)01249-4

[45] Bingol B, Wang CF, Arnott D, Cheng D, Peng J, Sheng M. Autophosphorylated CaMKIIalpha acts as a scaffold to recruit proteasomes to dendritic spines. Cell. 2010; 140: 567–578. doi: 10.1016/j.cell.2010.01.024 20178748 10.1016/j.cell.2010.01.024

[46] Ghislain M, Udvardy A, Mann C. *S*. *cerevisiae* 26S protease mutants arrest cell division in G2/metaphase. Nature. 1993; 366: 358–362. doi: 10.1038/366358a0 8247132 10.1038/366358a0

[47] Xu Q, Singer RA, Johnston GC. Sug1 modulates yeast transcription activation by Cdc68. Mol Cell Biol. 1995; 15: 6025–6035. 7565755 10.1128/mcb.15.11.6025 PMC230854

[48] Rubin DM, Glickman MH, Larsen CN, Dhruvakumar S, Finley D. Active site mutants in the six regulatory particle ATPases reveal multiple roles for ATP in the proteasome. EMBO J. 1998; 17: 4909–4919. doi: 10.1093/emboj/17.17.4909 9724628 10.1093/emboj/17.17.4909 PMC1170820

[49] Kim YC, DeMartino GN. C termini of proteasomal ATPases play nonequivalent roles in cellular assembly of mammalian 26 S proteasome. J Biol Chem. 2011; 286: 26652–26666. doi: 10.1074/jbc.M111.246793 21628461 10.1074/jbc.M111.246793 PMC3143629

[50] Glickman MH, Rubin DM, Fu H, Larsen CN, Coux O, Wefes I et al Functional analysis of the proteasome regulatory particle. Mol Biol Rep. 1999; 26: 21–28. 10363642 10.1023/a:1006928316738

[51] Russell SJ, Reed SH, Huang W, Friedberg EC, Johnston SA. The 19S regulatory complex of the proteasome functions independently of proteolysis in nucleotide excision repair. Mol Cell. 1999; 3: 687–695. 10394357 10.1016/s1097-2765(01)80001-0

[52] Ezhkova E, Tansey WP. Proteasomal ATPases link ubiquitylation of histone H2B to methylation of histone H3. Mol Cell. 2004; 13: 435–442. 14967150 10.1016/s1097-2765(04)00026-7

[53] Lee D, Ezhkova E, Li B, Pattenden SG, Tansey WP, Workman JL. The proteasome regulatory particle alters the SAGA coactivator to enhance its interactions with transcriptional activators. Cell. 2005; 123: 423–436. doi: 10.1016/j.cell.2005.08.015 16269334 10.1016/j.cell.2005.08.015

[54] Chouduri AU, Tokumoto T, Dohra H, Ushimaru T, Yamada S. Functional and biochemical characterization of the 20S proteasome in a yeast temperature-sensitive mutant, *rpt6-1*. BMC Biochem. 2008; 9: 20 doi: 10.1186/1471-2091-9-20 18644121 10.1186/1471-2091-9-20 PMC2515314

[55] Finley D, Ulrich HD, Sommer T, Kaiser P. The ubiquitin-proteasome system of *Saccharomyces cerevisiae*. Genetics. 2012; 192: 319–360. doi: 10.1534/genetics.112.140467 23028185 10.1534/genetics.112.140467 PMC3454868

[56] Glickman MH, Rubin DM, Fried VA, Finley D. The regulatory particle of the *Saccharomyces cerevisiae* proteasome. Mol Cell Biol. 1998; 18: 3149–3162. 9584156 10.1128/mcb.18.6.3149 PMC108897

[57] Tomko RJ, Funakoshi M, Schneider K, Wang J, Hochstrasser M. Heterohexameric ring arrangement of the eukaryotic proteasomal ATPases: implications for proteasome structure and assembly. Mol Cell. 2010; 38: 393–403. doi: 10.1016/j.molcel.2010.02.035 20471945 10.1016/j.molcel.2010.02.035 PMC2879271

[58] Ehlinger A, Park S, Fahmy A, Lary JW, Cole JL, Finley D et al Conformational dynamics of the Rpt6 ATPase in proteasome assembly and Rpn14 binding. Structure. 2013; 21: 753–765. doi: 10.1016/j.str.2013.02.021 23562395 10.1016/j.str.2013.02.021 PMC3670613

[59] Park S, Roelofs J, Kim W, Robert J, Schmidt M, Gygi SP et al Hexameric assembly of the proteasomal ATPases is templated through their C termini. Nature. 2009; 459: 866–870. doi: 10.1038/nature08065 19412160 10.1038/nature08065 PMC2722381

[60] Heinemeyer W, Kleinschmidt JA, Saidowsky J, Escher C, Wolf DH. Proteinase yscE, the yeast proteasome/multicatalytic-multifunctional proteinase: mutants unravel its function in stress induced proteolysis and uncover its necessity for cell survival. EMBO J. 1991; 10: 555–562. 2001673 10.1002/j.1460-2075.1991.tb07982.x PMC452684

[61] Teixeira LK, Reed SI. Ubiquitin ligases and cell cycle control. Annu Rev Biochem. 2013; 82: 387–414. doi: 10.1146/annurev-biochem-060410-105307 23495935 10.1146/annurev-biochem-060410-105307

[62] Bassermann F, Eichner R, Pagano M. The ubiquitin proteasome system—implications for cell cycle control and the targeted treatment of cancer. Biochim Biophys Acta. 2014; 1843: 150–162. doi: 10.1016/j.bbamcr.2013.02.028 23466868 10.1016/j.bbamcr.2013.02.028 PMC3694769

[63] McGurk L, Berson A, Bonini N. Drosophila as an In Vivo Model for Human Neurodegenerative Disease. Genetics. 2015; 201: 377–402. doi: 10.1534/genetics.115.179457 26447127 10.1534/genetics.115.179457 PMC4596656

[64] Mason RP, Giorgini F. Modeling Huntington disease in yeast: perspectives and future directions. Prion. 2011; 5: 269–276. doi: 10.4161/pri.18005 22052350 10.4161/pri.5.4.18005 PMC4012407

[65] Colby DW, Chu Y, Cassady JP, Duennwald M, Zazulak H, Webster JM et al Potent inhibition of huntingtin aggregation and cytotoxicity by a disulfide bond-free single-domain intracellular antibody. Proc Natl Acad Sci U S A. 2004; 101: 17616–17621. doi: 10.1073/pnas.0408134101 15598740 10.1073/pnas.0408134101 PMC539732

[66] Duennwald ML, Lindquist S. Impaired ERAD and ER stress are early and specific events in polyglutamine toxicity. Genes Dev. 2008; 22: 3308–3319. doi: 10.1101/gad.1673408 19015277 10.1101/gad.1673408 PMC2600758

[67] Petrucelli L, Dawson TM. Mechanism of neurodegenerative disease: role of the ubiquitin proteasome system. Ann Med. 2004; 36: 315–320. 15224658 10.1080/07853890410031948

[68] Chen Q, Ding Q, Keller JN. The stationary phase model of aging in yeast for the study of oxidative stress and age-related neurodegeneration. Biogerontology. 2005; 6: 1–13. doi: 10.1007/s10522-004-7379-6 15834659 10.1007/s10522-004-7379-6

[69] Kaeberlein M. Lessons on longevity from budding yeast. Nature. 2010; 464: 513–519. doi: 10.1038/nature08981 20336133 10.1038/nature08981 PMC3696189

[70] Adams J, Palombella VJ, Sausville EA, Johnson J, Destree A, Lazarus DD et al Proteasome inhibitors: a novel class of potent and effective antitumor agents. Cancer Res. 1999; 59: 2615–2622. 10363983

[71] Kumeda SI, Deguchi A, Toi M, Omura S, Umezawa K. Induction of G1 arrest and selective growth inhibition by lactacystin in human umbilical vein endothelial cells. Anticancer Res. 1999; 19: 3961–3968. 10628338

[72] Machiels BM, Henfling ME, Gerards WL, Broers JL, Bloemendal H, Ramaekers FC et al Detailed analysis of cell cycle kinetics upon proteasome inhibition. Cytometry. 1997; 28: 243–252. 9222110

[73] Kisselev AF, Goldberg AL. Proteasome inhibitors: from research tools to drug candidates. Chem Biol. 2001; 8: 739–758. 11514224 10.1016/s1074-5521(01)00056-4

[74] Kachroo AH, Laurent JM, Yellman CM, Meyer AG, Wilke CO, Marcotte EM. Evolution. Systematic humanization of yeast genes reveals conserved functions and genetic modularity. Science. 2015; 348: 921–925. doi: 10.1126/science.aaa0769 25999509 10.1126/science.aaa0769 PMC4718922

[75] Sokolova V, Li F, Polovin G, Park S. Proteasome Activation is Mediated via a Functional Switch of the Rpt6 C-terminal Tail Following Chaperone-dependent Assembly. Sci Rep. 2015; 5: 14909 doi: 10.1038/srep14909 26449534 10.1038/srep14909 PMC4598862

[76] Aufderheide A, Beck F, Stengel F, Hartwig M, Schweitzer A, Pfeifer G et al Structural characterization of the interaction of Ubp6 with the 26S proteasome. Proc Natl Acad Sci U S A. 2015; 112: 8626–8631. doi: 10.1073/pnas.1510449112 26130806 10.1073/pnas.1510449112 PMC4507206

[77] Sledz P, Unverdorben P, Beck F, Pfeifer G, Schweitzer A, Forster F et al Structure of the 26S proteasome with ATP-gammaS bound provides insights into the mechanism of nucleotide-dependent substrate translocation. Proc Natl Acad Sci U S A. 2013; 110: 7264–7269. doi: 10.1073/pnas.1305782110 23589842 10.1073/pnas.1305782110 PMC3645540

[78] Kim YC, Li X, Thompson D, DeMartino GN. ATP binding by proteasomal ATPases regulates cellular assembly and substrate-induced functions of the 26 S proteasome. J Biol Chem. 2013; 288: 3334–3345. doi: 10.1074/jbc.M112.424788 23212908 10.1074/jbc.M112.424788 PMC3561553

[79] Park S, Li X, Kim HM, Singh CR, Tian G, Hoyt MA et al Reconfiguration of the proteasome during chaperone-mediated assembly. Nature. 2013; 497: 512–516. doi: 10.1038/nature12123 23644457 10.1038/nature12123 PMC3687086

[80] Tian G, Park S, Lee MJ, Huck B, McAllister F, Hill CP et al An asymmetric interface between the regulatory and core particles of the proteasome. Nat Struct Mol Biol. 2011; 18: 1259–1267. doi: 10.1038/nsmb.2147 22037170 10.1038/nsmb.2147 PMC3210322

[81] Xie Y, Varshavsky A. UFD4 lacking the proteasome-binding region catalyses ubiquitination but is impaired in proteolysis. Nat Cell Biol. 2002; 4: 1003–1007. doi: 10.1038/ncb889 12447385 10.1038/ncb889

[82] Lin JT, Chang WC, Chen HM, Lai HL, Chen CY, Tao MH et al Regulation of feedback between protein kinase A and the proteasome system worsens Huntington's disease. Mol Cell Biol. 2013; 33: 1073–1084. doi: 10.1128/MCB.01434-12 23275441 10.1128/MCB.01434-12 PMC3623082

[83] Moloney TC, Hyland R, O'Toole D, Paucard A, Kirik D, O'Doherty A et al Heat shock protein 70 reduces alpha-synuclein-induced predegenerative neuronal dystrophy in the alpha-synuclein viral gene transfer rat model of Parkinson's disease. CNS Neurosci Ther. 2014; 20: 50–58. doi: 10.1111/cns.12200 24279716 10.1111/cns.12200 PMC6493192

[84] Wirz KT, Keitel S, Swaab DF, Verhaagen J, Bossers K. Early molecular changes in Alzheimer disease: can we catch the disease in its presymptomatic phase? J Alzheimers Dis. 2014; 38: 719–740. doi: 10.3233/JAD-130920 24072070 10.3233/JAD-130920

[85] Rodriguez-Arellano JJ, Parpura V, Zorec R, Verkhratsky A. Astrocytes in physiological aging and Alzheimer's disease. Neuroscience. 2016; 323: 170–182. doi: 10.1016/j.neuroscience.2015.01.007 25595973 10.1016/j.neuroscience.2015.01.007

[86] Lin YY, Lu JY, Zhang J, Walter W, Dang W, Wan J et al Protein acetylation microarray reveals that NuA4 controls key metabolic target regulating gluconeogenesis. Cell. 2009; 136: 1073–1084. doi: 10.1016/j.cell.2009.01.033 19303850 10.1016/j.cell.2009.01.033 PMC2696288

[87] Mitchell L, Huard S, Cotrut M, Pourhanifeh-Lemeri R, Steunou AL, Hamza A et al mChIP-KAT-MS, a method to map protein interactions and acetylation sites for lysine acetyltransferases. Proc Natl Acad Sci U S A. 2013; 110: E1641–E1650. doi: 10.1073/pnas.1218515110 23572591 10.1073/pnas.1218515110 PMC3637784

[88] Kikuchi J, Iwafune Y, Akiyama T, Okayama A, Nakamura H, Arakawa N et al Co- and post-translational modifications of the 26S proteasome in yeast. Proteomics. 2010; 10: 2769–2779. doi: 10.1002/pmic.200900283 20486117 10.1002/pmic.200900283

[89] Besche HC, Sha Z, Kukushkin NV, Peth A, Hock EM, Kim W et al Autoubiquitination of the 26S proteasome on Rpn13 regulates breakdown of ubiquitin conjugates. EMBO J. 2014;33: 1159–1176. doi: 10.1002/embj.201386906 24811749 10.1002/embj.201386906 PMC4193922

[90] Zhang F, Su K, Yang X, Bowe DB, Paterson AJ, Kudlow JE. O-GlcNAc modification is an endogenous inhibitor of the proteasome. Cell. 2003; 115: 715–725. 14675536 10.1016/s0092-8674(03)00974-7

