# Supplementary material for: Phosphorylation of the 19S regulatory particle ATPase subunit, Rpt6, modifies susceptibility to proteotoxic stress and protein aggregation
Source: PLoS One. 2017 Jun 29;12(6):e0179893. doi: 10.1371/journal.pone.0179893 (PMC5491056; doi:10.1371/journal.pone.0179893)
Supplement: S1 Fig — rpt6Δ null strains overexpressing RPT6 or rpt6-S120A were sensitive to ethanol 8%. All plasmids transformed in the experiment were 2μ. (PDF) [file pone.0179893.s001.pdf]

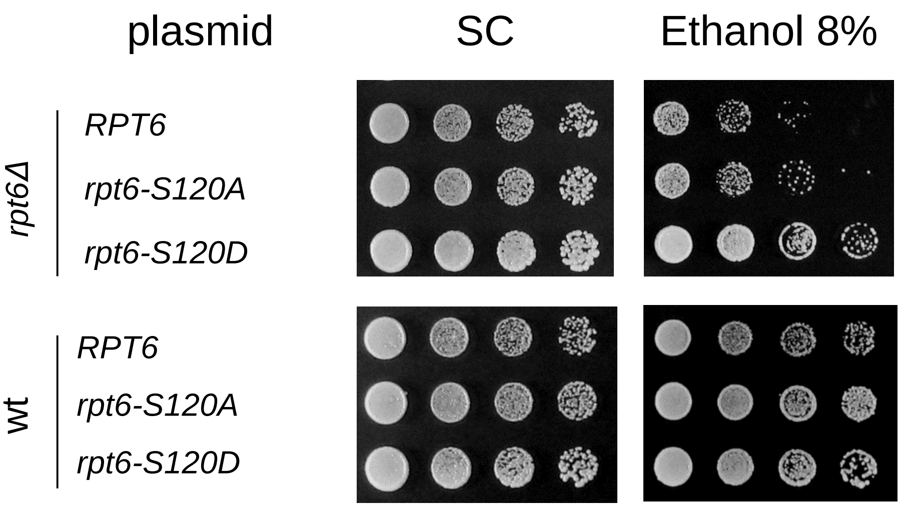

Figure S1. Overexpression of phosphomimetic *rpt6-S120D* promotes resistance to proteotoxic stress in the *RPT6* null background. *rpt6Δ* null strains overexpressing *RPT6* or *rpt6-S120A* were sensitive to ethanol 8%. All plasmids transformed in the experiment were 2µ.
