# Supplementary material for: Phosphorylation of the 19S regulatory particle ATPase subunit, Rpt6, modifies susceptibility to proteotoxic stress and protein aggregation
Source: PLoS One. 2017 Jun 29;12(6):e0179893. doi: 10.1371/journal.pone.0179893 (PMC5491056; doi:10.1371/journal.pone.0179893)
Supplement: S2 Fig — (A) Growth assays for all mutants challenged with elevated temperature or protein instability and DNA repair blocking agents. (B) WT and mutant strains were mated to WT yeast to produce diploids. Heterozygous diploid strains (*) had comparable growth to WT diploid yeast indicating that the rpt6 mutants are not dominant. (PDF) [file pone.0179893.s002.pdf]

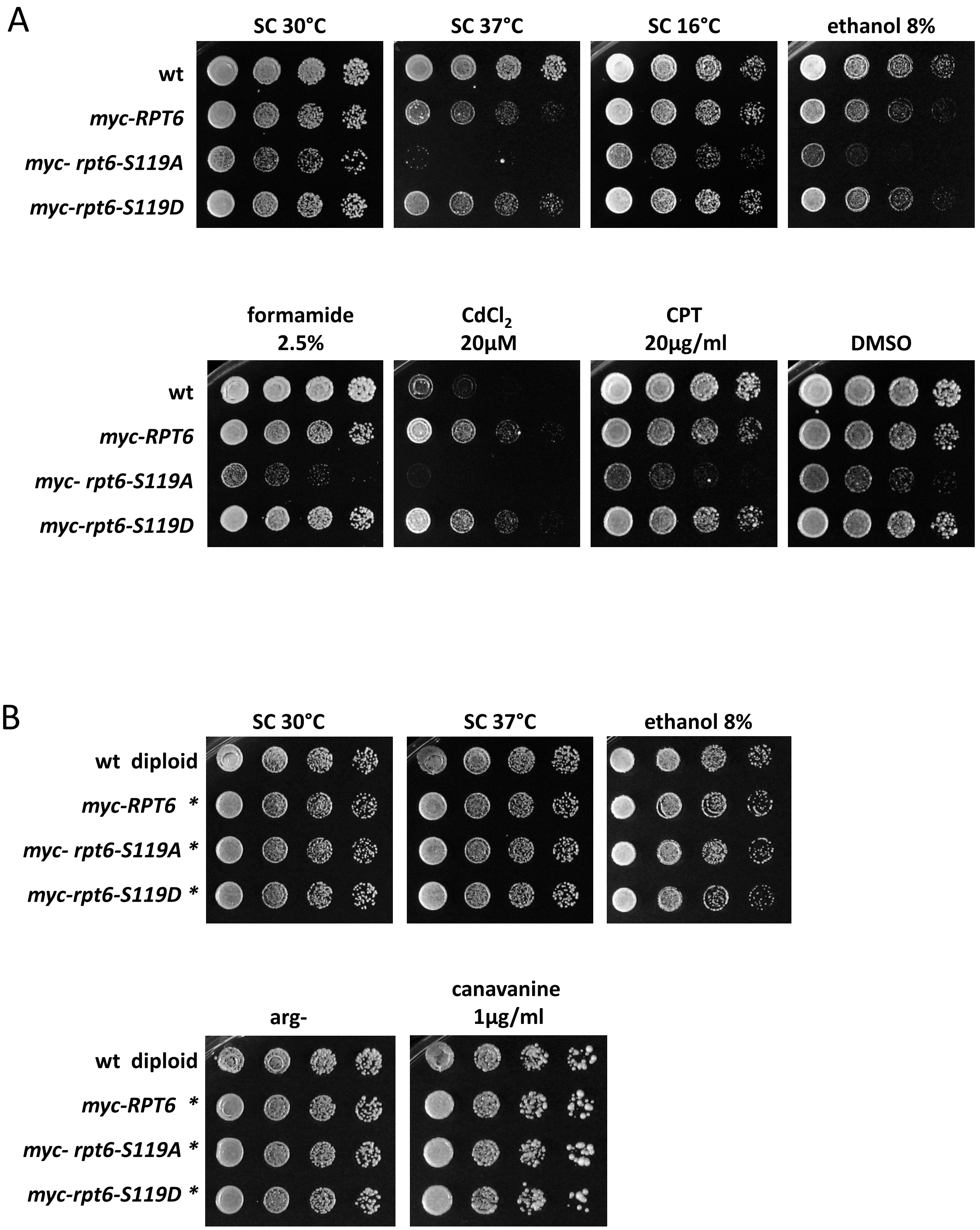

Figure S2. **The *rpt6-S119A* mutant had increased susceptibility to proteotoxic stress. (A)** Growth assays for all mutants challenged with elevated temperature or protein instability and DNA repair blocking agents. **(B)** WT and mutant strains were mated to WT yeast to produce diploids. Heterozygous diploid strains (*) had comparable growth to WT diploid yeast indicating that the *rpt6* mutants are not dominant.
