# Supplementary material for: Phosphorylation of the 19S regulatory particle ATPase subunit, Rpt6, modifies susceptibility to proteotoxic stress and protein aggregation
Source: PLoS One. 2017 Jun 29;12(6):e0179893. doi: 10.1371/journal.pone.0179893 (PMC5491056; doi:10.1371/journal.pone.0179893)
Supplement: S3 Fig — Flow cytometry analysis performed during logarithmic phase A600 0.6 (30°C and 34°C) and stationary phase A600 >5 (30°C) revealed a similar delay in cell cycle progression in all myc-tagged strains. (PDF) [file pone.0179893.s003.pdf]

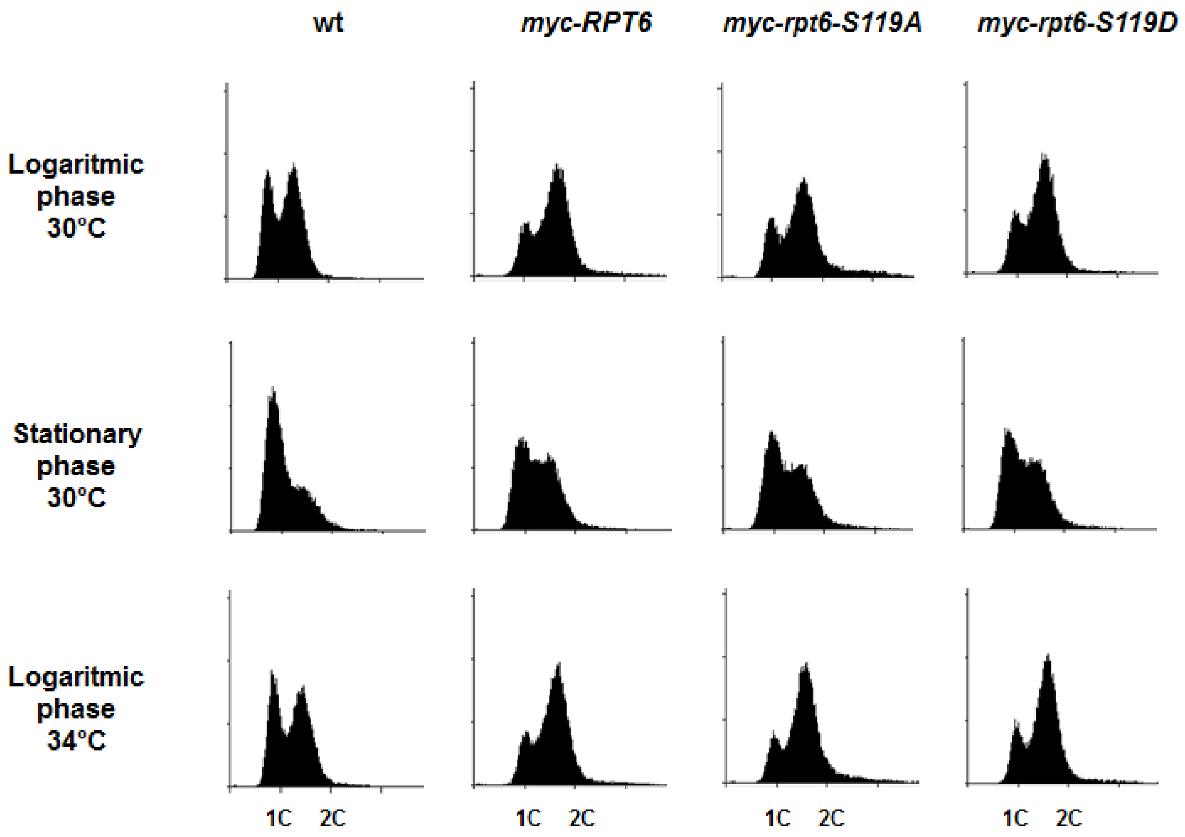

Figure S3. Cell cycle profiles of *myc-RPT6*, *myc-rpt6-S119A, and myc-rpt6-S119D* strains were comparable. Flow cytometry analysis performed during logarithmic phase A_{600}=0.6 (30°C and 34°C) and stationary phase A_{600}>5 (30°C) revealed a similar delay in cell cycle progression in all myc-tagged strains.
