# Supplementary material for: Phosphorylation of the 19S regulatory particle ATPase subunit, Rpt6, modifies susceptibility to proteotoxic stress and protein aggregation
Source: PLoS One. 2017 Jun 29;12(6):e0179893. doi: 10.1371/journal.pone.0179893 (PMC5491056; doi:10.1371/journal.pone.0179893)
Supplement: S4 Fig — Strains were grown in liquid ura- medium at 30°C and plated to asses viability after 1, 5, 7, 10, 12 and 15 days of growth. The graph is the average of three independent experiments performed with duplicate samples. Values were plotted relative to myc-RPT6 transformed with vector. (PDF) [file pone.0179893.s004.pdf]

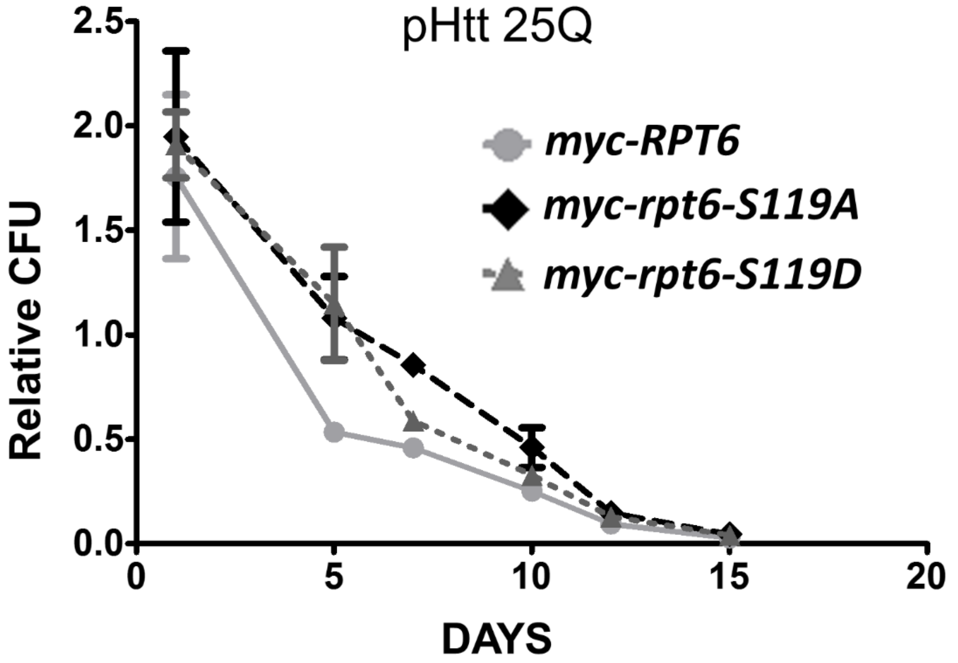

Figure S4. Strains *myc-rpt6-S119A* and *myc-rpt6-S119D* expressing Htt25Q have a similar loss of viability upon chronological aging. Strains were grown in liquid ura- medium at 30°C and plated to asses viability after 1, 5, 7, 10, 12 and 15 days of growth. The graph is the average of three independent experiments performed with duplicate samples. Values were plotted relative to *myc-RPT6* transformed with vector.
