# Supplementary material for: Phosphorylation of the 19S regulatory particle ATPase subunit, Rpt6, modifies susceptibility to proteotoxic stress and protein aggregation
Source: PLoS One. 2017 Jun 29;12(6):e0179893. doi: 10.1371/journal.pone.0179893 (PMC5491056; doi:10.1371/journal.pone.0179893)
Supplement: S1 Table — (PDF) [file pone.0179893.s005.pdf]

Table S1 Yeast strains

ID number	Genotype
LPY6496	*MAT* a *his3 Δ1 leu2Δ0 ura3Δ0* (BY4743)
LPY15916	BY diploid wild-type
LPY16270	BY MATα *rpt6Δ::kanMX* + pLP2636
LPY17011	BY *MAT* α *5'-RPT6 -kanMX-N9xMYC-RPT6-RPT6-3’*
LPY18040	BY *MAT* a MYC-*RPT6*
LPY18630	BY MATα *rpt6Δ::natMX* + pLP2636
LPY19188	BY *MAT* a *MYC-rpt6-S120A*
LPY19193	BY *MAT* a *MYC-rpt6-S120D*
