# Supplementary material for: Phosphorylation of the 19S regulatory particle ATPase subunit, Rpt6, modifies susceptibility to proteotoxic stress and protein aggregation
Source: PLoS One. 2017 Jun 29;12(6):e0179893. doi: 10.1371/journal.pone.0179893 (PMC5491056; doi:10.1371/journal.pone.0179893)
Supplement: S2 Table — (PDF) [file pone.0179893.s006.pdf]

Table S2 Plasmids

ID number	Plasmid	Source
pLP126	pRS316 (*URA3* , cen)	
pLP192	*Gal-Cre (URA3,* cen)	Life technologies
pLP196	*Gal-Cre (HIS3,* cen)	Life technologies
pLP1630	*TA::mx4::natR* switcher	Boone lab
pLP1956	*9MYC-KanMX*	Mark Trautwein
pLP2636	*RPT6* in pRS316 (1.8kb *Eco* RI-*Kpn* I fragment)	
pLP2855	*5´-RPT6-kanMX-N9xMYC-RPT6-RPT6-3'* in TOPO.	
pLP2858	*5´-RPT6-kanMX-N9xMYC-rpt6-S120A-RPT6-3'* in TOPO.	
pLP2859	*5´-RPT6-kanMX-N9xMYC-rpt6-S120D-RPT6-3'* in TOPO.	
pLP3012	*Htt-72Q-GFP* in pRS416	Addgene
pLP3013	*Htt-103Q-GFP* in pRS416	Addgene
pLP3014	*Htt-25Q-GFP* in pRS416	Addgene
