# Supplementary material for: Phosphorylation of the 19S regulatory particle ATPase subunit, Rpt6, modifies susceptibility to proteotoxic stress and protein aggregation
Source: PLoS One. 2017 Jun 29;12(6):e0179893. doi: 10.1371/journal.pone.0179893 (PMC5491056; doi:10.1371/journal.pone.0179893)
Supplement: S3 Table — (PDF) [file pone.0179893.s007.pdf]

Table S3 Oligonucleotides

ID number	Primer	Sequence
oLP236	KANMX-sense	GATGACGAGCGTAATGGCTG
oLP883	KANMX-antisense	CAGCCATTACGCTCGTCATC
oLP1707	RPT6cas-F	GAAGGGCCCGAGCGAGGTGGCAAAAGTTG
oLP1708	RPT6cas-R	GAACGGGCCCCCCGGCTT
oLP1751	RPT6-Ntag-F	CAGTAACAGTTAGGTTAGGTTTTATGTAAACGGAACAACAGTATAGTGGGATACTATCACGACAATGTGCAGGTCGACAACCCTTAAT
oLP1752	RPT6-Ntag-R	CTGTTCAAAATATGGTTTGATACCACTTTCGTGGGTTTCTAATACTATATTGGAGGATGTTACAGCAGCTGTGCGGCCGCATAGGCCACT
	RPT6-D308G-F	CACTTTTGAGACCCGGTAGAATAGATAGG
	RPT6-D308G-R	CCTATCTATTCTACCGGGTCTCAAAAGTG
	RPT6-S120A-F	GAGTTTGTCTAAGGAGTGACGCTTATATGTTGCATAAAGTTC
	RPT6-S120A-R	GAACTTTATGCAACATATAAGCGTCACTCCTTAGACAAACTC
	RPT6-S120D-F	GAGTTTGTCTAAGGAGTGACGATTATATGTTGCATAAAGTTC
	RPT6-S120D-R	GAACTTTATGCAACATATAATCGTCACTCCTTAGACAAACTC
